# MPX-004 and MPX-007: New Pharmacological Tools to Study the Physiology of NMDA Receptors Containing the GluN2A Subunit

**DOI:** 10.1371/journal.pone.0148129

**Published:** 2016-02-01

**Authors:** Robert A. Volkmann, Christopher M. Fanger, David R. Anderson, Venkata Ramana Sirivolu, Kathy Paschetto, Earl Gordon, Caterina Virginio, Melanie Gleyzes, Bruno Buisson, Esther Steidl, Susanna B. Mierau, Michela Fagiolini, Frank S. Menniti

**Affiliations:** 1 Mnemosyne Pharmaceuticals, Inc. (formerly Luc Therapeutics) 400 Technology Square, Cambridge, MA 02139, United States of America; 2 Jubilant Biosys Limited, #96, Industrial Suburb 2nd Stage, Yeshwantpur Bangalore - 560 022 Karnataka, India; 3 Jubilant Discovery Services, Inc. 365 Phoenixville Pike, Malvern, PA 19355, United States of America; 4 Aptuit Medicines Research Centre, Via Fleming 4, 37135 Verona, Italy; 5 Neuroservice, Domaine de Saint Hilaire, 595 rue Pierre Berthier, CS 30531–13593 Aix en Provence cedex 03, France; 6 FM Kirby Neurobiology Center, Boston Children’s Hospital, 300 Longwood Ave, Boston, MA 02115, United States of America; Creighton University, UNITED STATES

## Abstract

GluN2A is the most abundant of the GluN2 NMDA receptor subunits in the mammalian CNS. Physiological and genetic evidence implicate GluN2A-containing receptors in susceptibility to autism, schizophrenia, childhood epilepsy and neurodevelopmental disorders such as Rett Syndrome. However, GluN2A-selective pharmacological probes to explore the therapeutic potential of targeting these receptors have been lacking. Here we disclose a novel series of pyrazine-containing GluN2A antagonists exemplified by MPX-004 (5-(((3-chloro-4-fluorophenyl)sulfonamido)methyl)-N-((2-methylthiazol-5-yl)methyl)pyrazine-2-carboxamide) and MPX-007 (5-(((3-fluoro-4-fluorophenyl)sulfonamido)methyl)-N-((2-methylthiazol-5-yl)methyl)methylpyrazine-2-carboxamide). MPX-004 and MPX-007 inhibit GluN2A-containing NMDA receptors expressed in HEK cells with IC_50_s of 79 nM and 27 nM, respectively. In contrast, at concentrations that completely inhibited GluN2A activity these compounds have no inhibitory effect on GluN2B or GluN2D receptor-mediated responses in similar HEK cell-based assays. Potency and selectivity were confirmed in electrophysiology assays in Xenopus oocytes expressing GluN2A-D receptor subtypes. Maximal concentrations of MPX-004 and MPX-007 inhibited ~30% of the whole-cell current in rat pyramidal neurons in primary culture and MPX-004 inhibited ~60% of the total NMDA receptor-mediated EPSP in rat hippocampal slices. GluN2A-selectivity at native receptors was confirmed by the finding that MPX-004 had no inhibitory effect on NMDA receptor mediated synaptic currents in cortical slices from *GRIN2A* knock out mice. Thus, MPX-004 and MPX-007 offer highly selective pharmacological tools to probe GluN2A physiology and involvement in neuropsychiatric and developmental disorders.

## Introduction

Neurons that utilize glutamate as neurotransmitter comprise the core architecture of the brain. Glutamate synaptic transmission mediates information flow within this core network, and coordinates regulatory GABAergic, aminergic, and cholinergic networks [1]. Glutamate synapses have 3 types of ionotropic receptors, AMPA, KA, and NMDA [2], and a family of metabotropic receptors (mGluRs) [3]. AMPA receptors are the essential elements mediating fast excitatory transmission, whereas KA and mGluRs are primarily involved in pre- and post-synaptic modulatory functions. NMDA receptors mediate slow excitatory synaptic transmission, playing a key role in the integration of synaptic inputs. Perhaps more importantly, NMDA receptors regulate the strength of glutamate synapses [4] by promoting the insertion or removal of AMPA receptors in response to the strength and timing of pre- and post-synaptic activity [5]. This glutamate synaptic plasticity is a principal molecular mechanism for modifying the informational content and flow in glutamatergic neuronal networks. Thus, NMDA receptors may be considered a ‘master switch for learning and memory’ and provide a key therapeutic target for treatment of neuropsychiatric disease [6–10].

The NMDA receptor is a tetramer consisting of 2 GluN1 subunits and 2 GluN2 subunits, arranged as a dimer of GluN1/GluN2 dimers [11, 12]. The GluN1 subunit is encoded by a single gene with 8 splice variants, whereas there are 4 GluN2 subunits, GluN2A-D, that are individually coded [13, 14]. Each subunit is comprised of 4 modules: a ligand binding domain (LBD), a transmembrane domain (TMD) that forms the ion channel pore, an amino terminal domain (ATD) that serves a modulatory function, and an intracellular c-terminal domain (CTD) involved in anchoring the receptors to intracellular scaffolds and signaling complexes [2, 11, 12]. The ligand for the GluN1 subunit is glycine or D-serine, whereas that for the GluN2 subunits is glutamate. Once glycine or D-serine is bound to the GluN1 subunit, synaptically released glutamate binds to the GluN2 subunit, leading to NMDA receptor channel gating. The GluN2 subtype composition of NMDA receptors confers specific physiological characteristics including differences in glutamate and glycine affinities, channel kinetics, and interaction with allosteric modulators and intracellular complexes [6, 15, 16]. Forebrain principal neurons and striatal projection neurons express primarily GluN2A and GluN2B homomers and GluN2A/GluN2B heteromers [13, 17]. GluN2C- and GluN2D-containing receptors are expressed along with GluN2A and GluN2B in forebrain interneurons, and GluN2C is highly expressed in cerebellum [13, 17].

**Fig 1:**
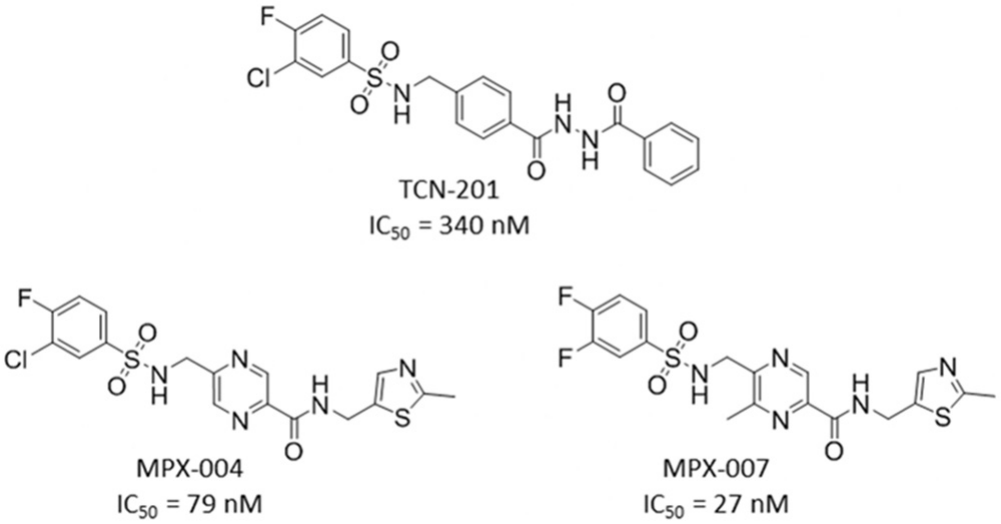
The structures of TCN-201, MPX-004 and MPX-007. The values are IC_50_s for inhibition of Ca^2+^ responses mediated by GluN2A receptors expressed in HEK cells.

There is a rich pharmacology of NMDA receptor modulators that have been essential in the investigation of the physiology of these receptors and their involvement in central nervous system disease [2, 18–21]. These include a variety of channel blockers as well as glutamate- or glycine-binding site antagonists [2]. There is one well developed class of subtype-selective compounds, the GluN2B negative allosteric modulators (NAMs) [22, 23]; however, until recently there have been few pharmacological tools to probe the physiology that is unique to receptors containing the other GluN2 subunits, A, C or D [18, 19]. In 2010, Bettini and coworkers [24] disclosed a selective GluN2A receptor antagonist (3-chloro-4-fluoro-N-[4-[[2-(phenylcarbonyl)hydrazino]carbonyl]benzyl]benzenesulfonamide; TCN-201; Fig 1). While highly selective for inhibition of receptors containing GluN2A subunits over GluN2B subunits, this compound has inherent properties that limit its overall potential for characterization of GluN2A pharmacology in native systems. Thus, we undertook a medicinal chemistry optimization campaign to overcome these liabilities and create new tools for investigating GluN2A physiology. Beginning with the TCN-201 scaffold, we created more potent and soluble antagonists that maintained high selectivity for inhibition of GluN2A. We identified more drug-like molecules by eliminating of the hydrazide moiety, reducing the number of its H-bond donors and lowering lipophilicity. Here we describe a series of compounds that are highly potent and selective for inhibition of NMDA receptors containing GluN2A subunits, exemplified by MPX-004 and MPX-007 (Fig 1).

## Results

To investigate the structure-activity relationships for new TCN-201 analogs, we utilized 384-well plate-based assays employing HEK cells expressing human GluN2A, B, and D. NMDA receptor activity was assessed based on the increase in intracellular Ca^2+^ concentration induced by glutamate plus glycine, and monitored using the Ca^2+^ sensitive fluorescent dye fluo-8, in experiments similar to those described previously by others [25]. The potency and efficacy of standard NMDA receptor antagonists in these assays are similar to those reported in the literature ([2]; Table 1). Note that we screened at glutamate and glycine concentrations (3 μM) that produced maximal Ca^2+^ responses in each of these cell lines.

**Table 1:** Potency of standard NMDA antagonists for inhibition of Ca^2+^ responses mediated by GluN2A, GluN2B and GluN2D expressed in HEK cells.

Compound	GluN2A	GluN2B	GluN2D
	IC_50_ (nM) ± SEM (n)	IC_50_ (nM) ± SEM (n)	IC_50_ (nM) ± SEM (n)
**MK-801**	205 ± 26 (7)	75 ± 4 (2)	87 ± 3 (5)
**ARL-15896**	ND	510,000 ± 240,000 (3)	25,000 ± 8,000 (3)
**Ro 25–6981**	>10,000 (3)	20 ± 1 (58)	>10,000 (4)
**CP-101,606**	>10,000 (4)	16.2 ± 0.7 (57)	>10,000 (2)
**5,7-DCKA**	1170 ± 230 (34)	8400 ± 1400 (13)	>10,000 (3)
**PPDA**	4860 ± 960 (6)	8490 ± 1000 (6)	34,800 ± 8,800 (4)

Cells were stimulated with glutamate and glycine (3 μM each) in the presence of compounds at a range of concentrations. IC_50_ for inhibition of the Ca^2+^ response was fitted to the Hill equation using CDD Vault (Collaborative Drug Discovery, Burlingame, CA). Values are the mean IC_50_ ± SEM with the number of replicate curves indicated in parentheses. MK-801 and ARL-15896- subtype non-selective NMDA receptor channel blockers; Ro 25–6981 and CP-101,606- GluN2B-selective negative allosteric modulators; 5,7-DCKA- subtype non-selective glycine-site competitive antagonist [2]. ND- not determined.

### Structure activity relationships for TCN-201 analogs

**Fig 2:**
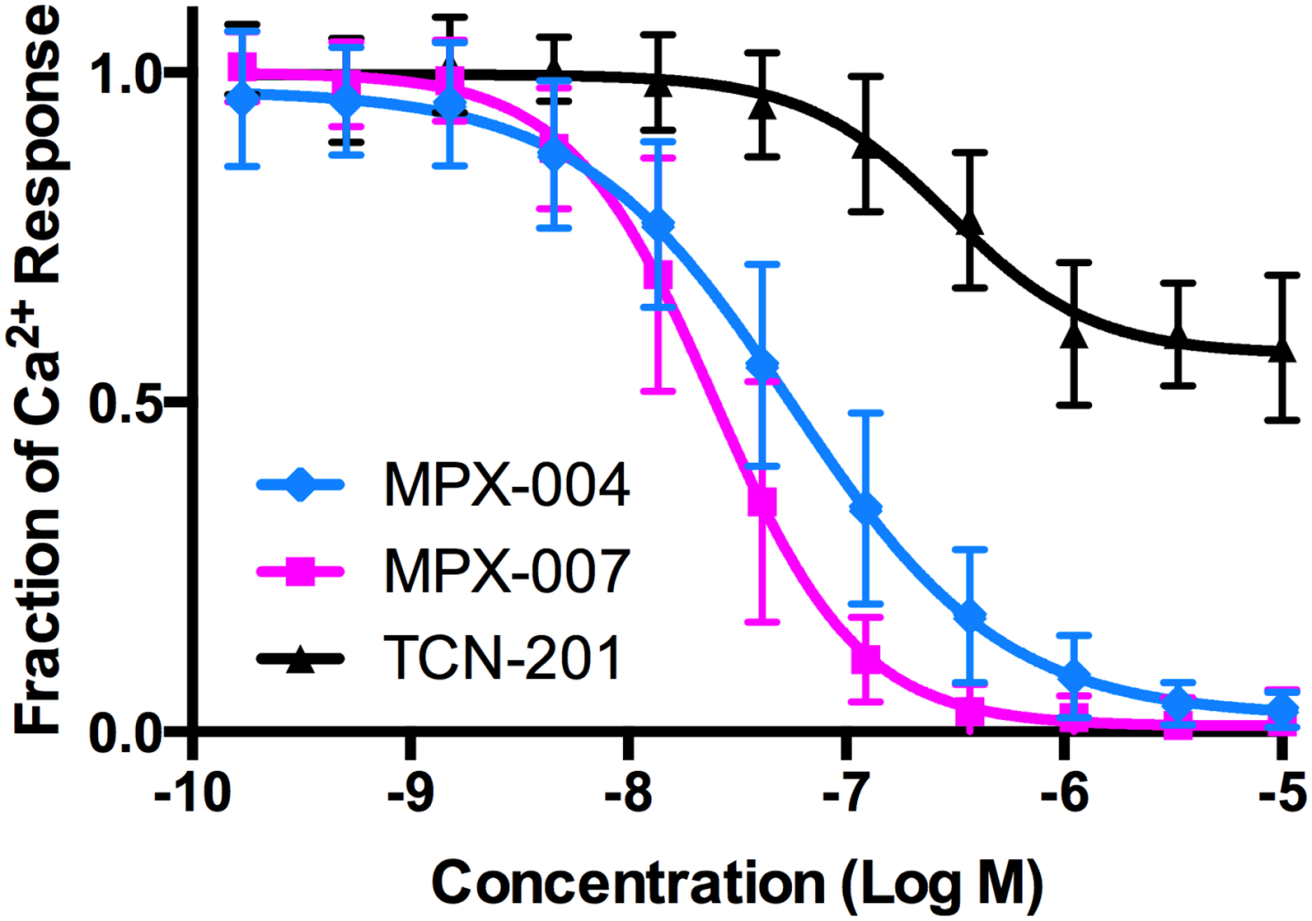
Concentration-response of TCN-201, MPX-004, and MPX-007 inhibition of Ca^2+^ responses mediated by GluN2A expressed in HEK cells. Cells were stimulated with glutamate and glycine (3 μM each) in the presence of compounds at a range of concentrations. Curves for inhibition of the Ca^2+^ response in GluN2A-expressing cells were derived from fits to the Hill equation using GraphPad Prism (v6.00 for Mac, GraphPad Software, La Jolla California USA). Whereas MPX-004 and MPX-007 achieve full inhibition of the GluN2A Ca^2+^ response by ~ 3 μM, TCN-201 never inhibits more than ~40% of the response. Each data point is a mean (± standard deviation) of data from 20–86 experiments).

**Fig 3:**
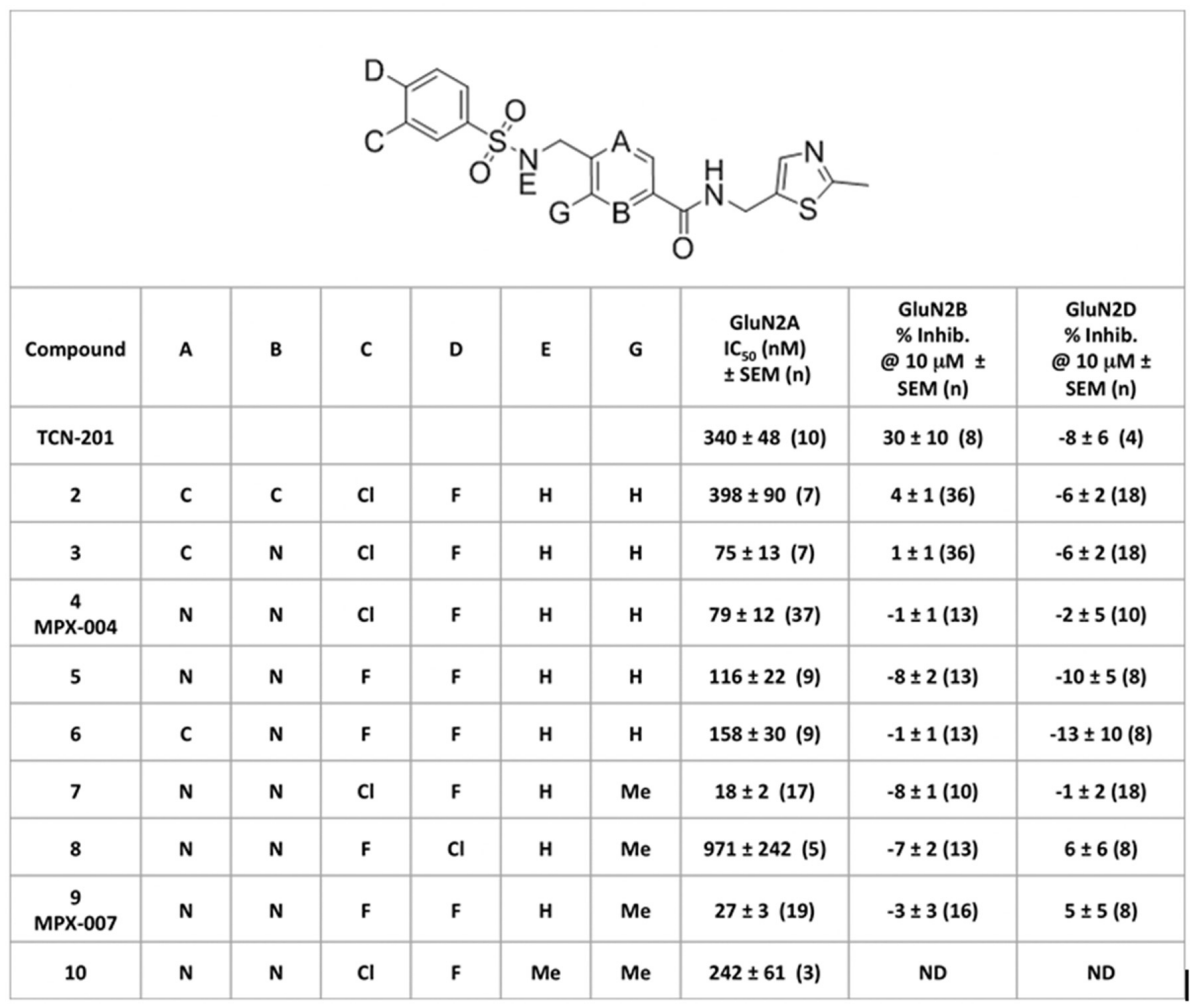
Potency or efficacy of TCN-201 and selected analogs for inhibition of Ca^2+^ responses mediated by GluN2A, GluN2B and GluN2D expressed in HEK cells. **C**ells were stimulated with glutamate and glycine (3 μM each) in the presence of compounds at a range of concentrations. IC_50_ for inhibition of the Ca^2+^ response in GluN2A-expressing cells was fitted to the Hill equation using CDD Vault. For GluN2B or GluN2D no IC_50_ could be determined, so the effect of each compound at 10 μM is shown as the % inhibition of the Ca^2+^ response (note that negative % inhibition represents an increase in Ca^2+^ response over glutamate plus glycine alone). Values are the mean IC_50_ or % ± SEM with the number of replicate curves indicated in parentheses. ND- not determined.

In our GluN2A screening assay, the potency of TCN-201 was relatively low (IC_50_ of 340 ± 48 nM; Fig 1). This may be due, at least in part, to functional competition with glycine at the high concentration used in our assay ([26], and see Discussion). TCN-201 also failed to achieve complete inhibition of GluN2A activity in this assay (maximum inhibition of 43 ± 3%; Fig 2). We believe this to be due to poor solubility in physiological solutions, limiting the ability to achieve concentrations that may have resulted in a greater degree of inhibition. As expected [24, 26], TCN-201 had less inhibitory activity against Ca^2+^ responses in HEK cells expressing GluN2B or GluN2D receptors in similar assays (Fig 3). However, we note we could not fully evaluate the selectivity of this compound in these HEK cell assays due to its poor solubility. Given the limitations of TCN-201 evident in our screening assays, we sought to create new analogs with increased potency and with solubility sufficient to fully evaluate inhibition of GluN2A and selectivity for GluN2A subunit containing receptors. Few phenylsulfonamide bioisosteres exist in the literature and, for that reason, we kept this feature of TCN-201 in our target molecules and concentrated on alterations involving the rest of the molecule. Here we report the identification of a new series of selective GluN2A receptor antagonists that met our objectives, exemplified by compounds **2–10** (Fig 3).

We initially determined that the hydrazide moiety of TCN-201 was not crucial for GluN2A inhibitory activity and that by replacing its phenyl hydrazide moiety with 2-(methylthiazol-5-yl)methanamine, selective GluN2A antagonists could be found (e.g., compound **2**). To lower the cLogP of analogs in this new series, carbon atoms in the central aromatic phenyl ring were replaced with nitrogen atoms. Pyridines **3** & **6** containing a single nitrogen atom provided more potent GluN2A inhibitory activity, whereas incorporation of two nitrogen atoms in the form of pyrazines (**4** and **5**) maintained increased inhibitory potency while improving ADME properties (solubility, efflux, HLM stability). Addition of a methyl group to the pyrazine nucleus further improved potency, with pyrazines **7** and **9** having IC_50_’s approximately a order of magnitude lower than TCN-201. Interestingly, switching the chlorine and fluorine substituents on the phenylsulfonamide aromatic ring resulted in significant loss of inhibitory potency (compare **7** and **8**), as did N-methylation of the phenyl sulfonamide moiety (compare **7** and **10**). Thus, structure-activity relationships for inhibition of GluN2A within this series are narrow but well defined. Compounds **2–10** at concentrations of 10 μM inhibited GluN2A activity more than 95% in the HEK cell assay, in contrast to TCN-201. None of the analogs in this series inhibited GluN2B or GluN2D activity more than 6.5% at 10 μM in similar HEK cell-base assays, indicating that GluN2A selectivity was largely maintained. In all, over 200 analogs [27] were synthesized and screened and from these we selected MPX-004 and MPX-007 for more in depth analysis. Both MPX-004 and MPX-007 are pyrazines with optimal halogen substituents on the phenylsulfonamide aryl ring. Both compounds are more potent than TCN-201 and completely block GluN2A-mediated Ca^2+^ responses in the HEK cell assay (Fig 2). MPX-007 is the more potent of the two compounds, owing to the addition of a methyl group to the pyrazine nucleus. These compounds also have improved physiochemical and ADME properties compared to TCN-201 (Table 2).

**Table 2:** Physical/Chemical and *in vitro* ADME properties of TCN-201, MPX-004 and MPX-007.

Compound	logP	H-bond donors	PSA	Solubility (μM)	HLM t½ (min)	MDCKA-B, B-A	Efflux ratio
**TCN-201**	3.39	3	104.4	8	19	7.5, 58.9	7.8
**MPX-004**	1.02	2	113.9	16	53	17.3, 66.1	3.8
**MPX-007**	0.69	2	113.9	68	63	14.2, 76.8	5.4

logP, hydrogen(H)-bond donors, and polar surface area (PSA) are parameters calculated using CDD Vault. Solubility is the concentration of the compound determined to be in solution by HPLC after dissolution in DMSO and then addition to saline. Note that for assay in biological systems the compounds were dissolved in 100% DMSO prior to dilution in assay buffers and greater final concentrations were achieved. Stability in human liver microsomes (HLM) is reported as the half-life (t½ in minutes). Permeability across MDCK cell monolayers was determined after compound was added on the side of the apical (A-B) or basal (B-A) membrane. The efflux ratio was calculated as the (A-B)/(B-A) permeability ratio as an index of the likelihood that compound were be excluded from the brain by active transport at the blood-brain barrier.

### Effects on NMDA receptor mediated currents for receptors expressed in Xenopus oocytes

**Fig 4:**
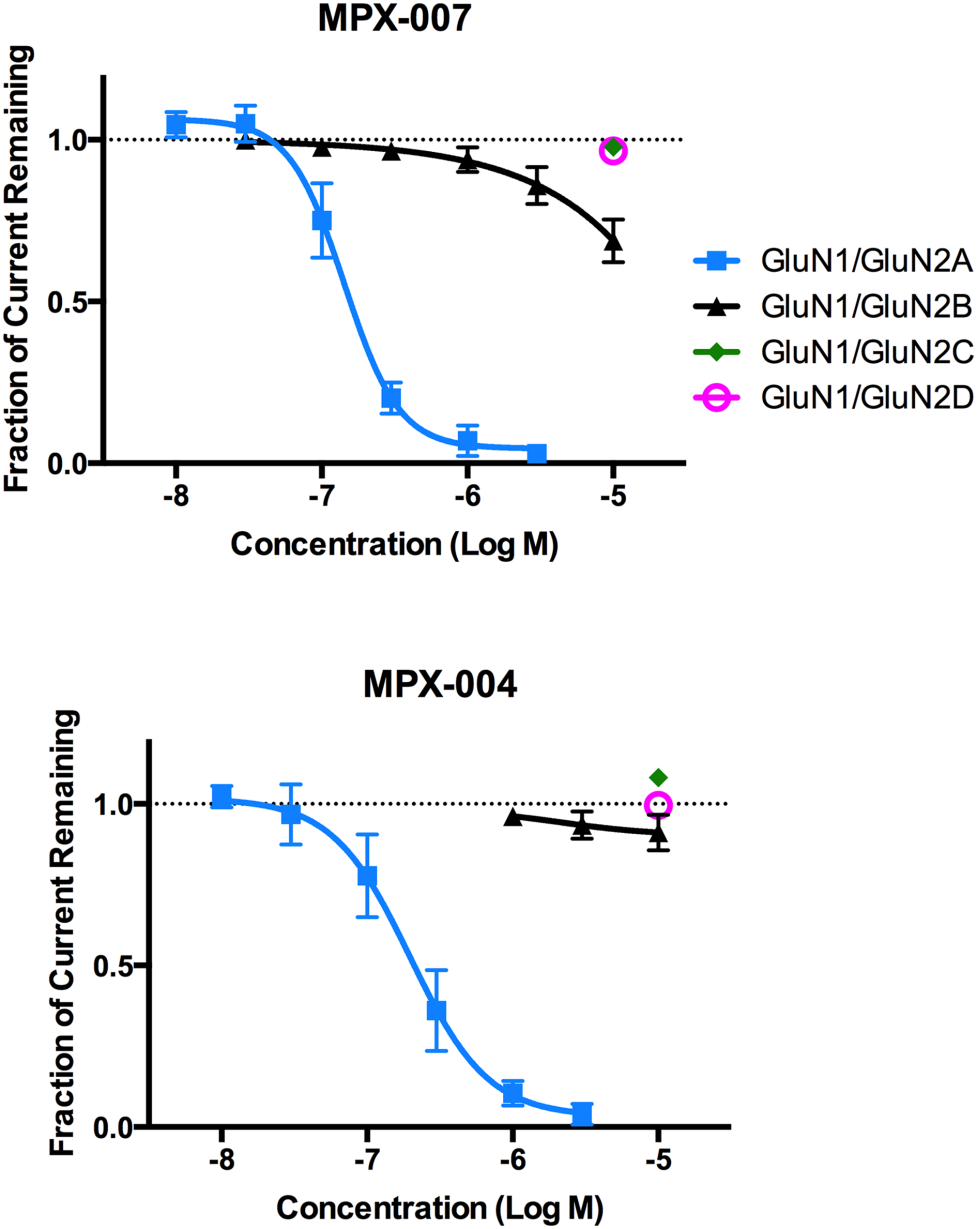
Effect of MPX-004 and MPX-007 on glutamate/glycine-induced currents in *Xenopus* oocytes expressing GluN1 and GluN2A, B, C, or D. Oocytes were exposed to MPX-004 or MPX-007 at concentrations from 10 nM to 10 μM as indicated. Inhibition curves were generated using GraphPad Prism and IC_50_ values were generated using CCD Vault. The IC_50_s for inhibition of GluN2A-mediated currents were 198 ± 17 and 143 ± 10 nM for MPX-004 and MPX-007, respectively. Each data point is a mean (± standard deviation) of data from 4–12 oocytes.

We evaluated MPX-004 and MPX-007 inhibition of NMDA receptor-mediated currents in *Xenopus* oocytes expressing human GluN1 + GluN2A. IC_50_s were 198 ± 17 nM (n = 12) for MPX-004 and 143 ± 10 nM (n = 4) for MPX-007 (IC_50_s ± SEM; Fig 4). These IC_50_s are slightly higher than those determined for GluN2A in the HEK cell assays. Slight reduction in potency in oocyte expression systems relative to mammalian expression systems is not uncommon, as discussed by others [28]. At concentrations up to 10 μM, MPX-004 only weakly (up to 8%) inhibited currents in oocytes expressing GluN2B, C, or D receptors (Fig 4) or in control oocytes (data not shown). MPX-007 at 10 μM was similarly ineffective for inhibition of GluN2C or D currents (Fig 4) and had no effect in control oocytes (data not shown). However, MPX-007 evidenced weak but clearly concentration-dependent inhibition of GluN2B mediated currents, blocking ~30% of current at 10 μM. This effect was not observed in the Ca^2+^/fluorescence assays using HEK cells. Arbitrarily assigning IC_50_s for GluN2B, C, and D as >30 μM for MPX-004 and >10 μM for MPX-007, these compounds are estimated to be at least 150- and 70-fold selective, respectively, for the GluN2A subtype over the other subtypes based on IC_50_ ratios. However, we anticipate these antagonists will be used in some experimental situations to achieve complete but selective GluN2A inhibition. Thus, although MPX-004 is less potent and soluble than MPX-007, it has a slight but potentially meaningful greater selectivity for GluN2A over GluN2B.

As a control, the small endogenous currents (~0.02–0.05 μA) found in uninjected oocytes were examined for potential inhibition by MPX-004 and MPX-007. No inhibition was observed; only an apparent slight current increase that was <1% of the current observed in GluN-expressing oocytes treated with glutamate + glycine.

### Lack of potent activity at other CNS-relevant targets

MPX-004 was screened at 1 μM for inhibition of binding of ligands to a panel of receptors and enzymes relevant to CNS pharmacology (CEREP-80). At this concentration, MPX-004 inhibited 5-HT1B antagonist binding by 35%, 5-HT2A agonist binding by 31% and EP4 agonist binding by 27%. Effects on the binding of ligands in the remainder of the panel were less than 25%. MPX-004 had no effect on AMPA receptor-mediated synaptic currents of pyramidal neurons in slices from mouse visual cortex- currents in the presence of 50 μM MPX-004: 118±21 pA, (n = 10), controls: 118±16 pA (n = 11).

### Effects on NMDA receptor-mediated responses in rat cortical neurons in primary culture

**Fig 5:**
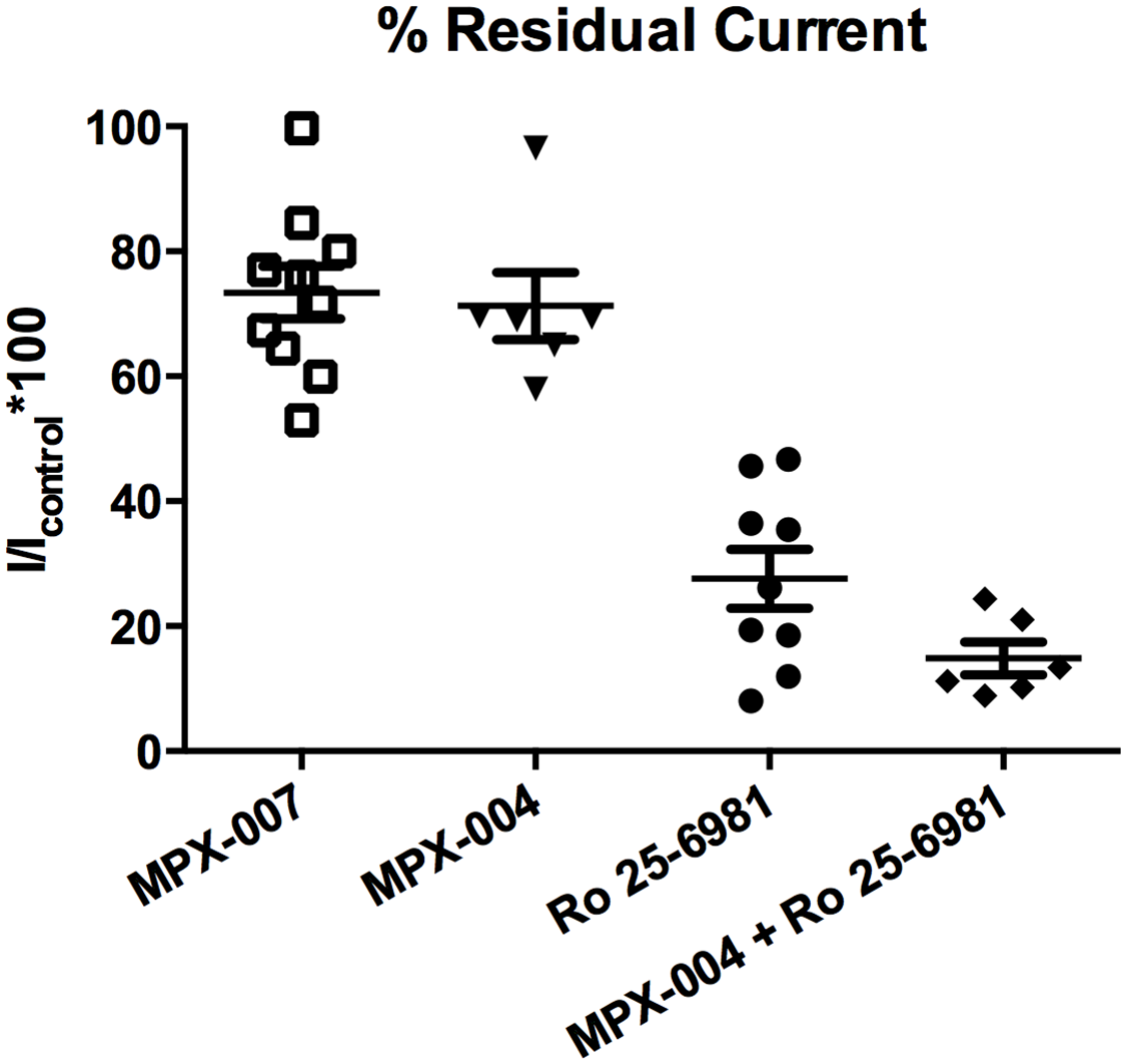
Effects of MPX-004 and MPX-007 on NMDA/glycine-induced currents of rat cortical neurons in primary culture. Cortical neurons were maintained in primary culture for 13–15 days and then examined using whole-cell manual patch clamp for current evoked by NMDA (100 μM) + glycine (10 μM) applied for 4 seconds during 10 second pulses to +20 or +40 mV from a holding potential of -70 mV. Inhibition of NMDA-activated currents was quantified during exposure to 10 μM MPX-007, MPX-004, Ro 25–6981, or a combination of Ro 25–6981 + MPX-004. Currents were blocked ~25–30% by either MPX-007 or MPX-004 alone, ~70% by Ro 25–6981 alone, and ~85% by Ro 25–6981 plus MPX-004.

Cortical neurons isolated from E18 rat embryos and cultured for two weeks were examined in whole-cell manual patch clamp experiments. During voltage steps to +40 mV, NMDA (100 μM) + glycine (10 μM) evoked currents were blocked (means ± SEMs) 29 ± 5% (n = 6 cells) and 27 ± 4% (n = 10) by MPX-004 or MPX-007, respectively (Fig 5). Currents in these neurons were blocked 72 ± 5% (n = 9) by a GluN2B NAM (Ro 25–6981; [29]) and 85 ± 3% (n = 6) by the combination of MPX-004 and Ro 25–6981. Residual currents not blocked by MPX-004 + Ro 25–6981 may yet be due to activity of GluN2B receptors, which are not completely blocked by GluN2B-selective NAMs such as Ro-25-6981 ([29] and data not shown). These data may be interpreted to indicate that in these cultures NMDA receptor responses are mediated by a roughly 70%/30% mix of GluN2B and GluN2A subunit-containing receptors.

### Effects on NMDA receptor-mediated responses in brain slice preparations

**Fig 6:**
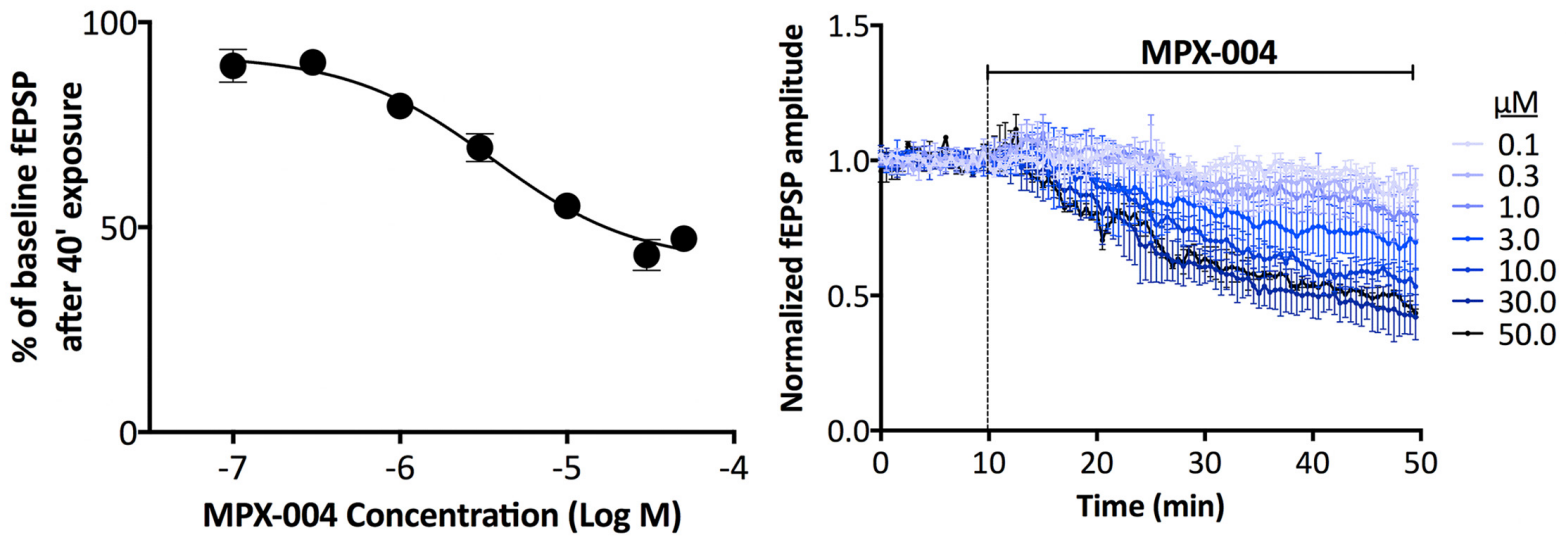
Effect of MPX-004 on isolated NMDA receptor-mediated fEPSPs in rat hippocampal CA1 stratum radiatum in response to stimulation of Schaffer collateral input. Slices were exposed to MPX-004 from 100 nM to 30 μM in half log concentration increments as well as to 50 μM. Right panel- Time course for inhibition of fEPSPs after application of different concentrations of MPX-004. Left panel- Percent inhibition at 40 min after application for each MPX-004 concentration. Maximum inhibition was approximately 60% of the fEPSPs at 30 or 50 μM. The IC_50_ of MPX-004 for inhibition of fEPSPs was 3.4 μM. Curves in the right panel and data point in the left panel are a mean (± SEM) of 4, 5, 6, 8, 6, 5 and 2 slices obtained from 2, 2, 3, 3, 2, 2 and 1 rats exposed to 0.1, 0.3, 1, 3, 10, 30 and 50 μM MPX-004, respectively. In separate experiments, a highly selective GluN2B NAM inhibited approximately 40% of the fEPSP (data not shown).

For these experiments, MPX-004 was used based on a slightly higher GluN2A selectivity compared to MPX-007. In hippocampal slices prepared from brains of 3- to 4-week old rats, MPX-004 caused a concentration-dependent reduction in NMDA receptor-mediated fEPSPs in region CA1 in response to Schaffer collateral stimulation (Fig 6). Maximum inhibition was approximately 60%, based on the fact that both 30 and 50 μM MPX-004 produced this same level of inhibition (normalized fractional fEPSP amplitudes of 0.42 ± 0.04 and 0.44 ± 0.04 at 40 min after incubation with 30 or 50 μM MPX-004, respectively). Application of D-AP5 at the conclusion of each experiment completely blocked the fEPSCs, demonstrating that they were mediated entirely through NMDA receptors. Furthermore, a maximally effective concentration of a highly specific GluN2B NAM inhibited the NMDA receptor-mediated fEPSPs by approximately 40% (normalized fractional fEPSP amplitude was of 0.59 ± 0.04 at 40 min after incubation with 30 μM GluN2B NAM). Together, these data may be interpreted to suggest that the 60% maximal inhibition observed with MPX-004 represents near complete inhibition of the GluN2A component of the NMDA receptor fEPSP in this preparation.

We next investigated whether MPX-004 was selective for native NMDA receptors containing the GluN2A subunit by testing the effect on *GRIN2A* KO mice. These mice, which lack NMDA receptors containing the GluN2A subunit, maintain a significant GluN2B-mediated NMDA receptor response at synapses from layer 4 onto layer 2/3 pyramidal cells in visual cortical slices at postnatal day (P)30 (Ref [30]). While MPX-004 significantly reduced the ratio of synaptic currents mediated by NMDA to those mediated by AMPA receptors in slices from WT, there was no significant difference (p~0.98, Student’s t-test) in the NMDAR/AMPAR ratio in slices from *GRIN2A* KO mice incubated with or without 50 μM MPX-004 (Table 3).

**Table 3:** Effect of MPX-004 on the ratio of NMDA receptor- to AMPA receptor-mediated synaptic currents (NMDAR/AMPAR ratio) at layer 4-to-2/3 synapses in mouse visual cortex.

	NMDAR/AMPAR ratio ± SEM (n)	
**Treatment**	**WT**	***GRIN2A* KO**
**Vehicle**	0.96 ± 0.07 (10)	0.77 ± 0.08 (5)
**MPX-004**	0.65 ± 0.10* (10)	0.76 ± 0.06 (3)

Acute slices from P28-31 WT or *GRIN2A* KO mice were incubated with 50 μM MPX-004 or vehicle for at least 40 minutes prior to recording. MPX-004 caused a statistically significant, ~30% reduction in the NMDAR/AMPAR ratio in slices from WT mice (* p~0.01, one-way ANOVA), but had no effect in slices from *GRIN2A* KO mice.

## Discussion

Bettini et al. [24] first disclosed a limited series of antagonists highly selective for NMDA receptors containing a GluN2A subunit. One of these compounds, TCN-201, has been used in several studies [31–33] to probe the role of GluN2A receptors in neurophysiological processes. However, in our hands, this compound was found to have significant limitations. Thus, we undertook a medicinal chemistry campaign to identify new analogs that maintained the selectivity of TCN-201 for GluN2A subunit containing NMDA receptors but with improved potency and solubility to serve as more effective pharmacological probes. From a series that comprised over 200 analogs [27], we identified MPX-004 and MPX-007 for characterization. Both compounds have meaningfully greater potency and solubility than TCN-201. These improvements are exemplified by the fact that both MPX analogs were amenable to analysis at concentrations sufficient to completely inhibit GluN2A activity and to evaluate the selectivity over other GluN2 subtypes in HEK cell and oocyte assays. We also note that the MPX analogs have improved drug-like properties over TCN-201. These include lack of the hydrazine core, lower logP, and reduction in H bond donors from 3 to 2. Unfortunately, these compounds retain a high polar surface area and evidence for an efflux liability in MDCK cell assays, characteristics that are not conducive to brain access after systemic administration. In fact, in preliminary studies, both compounds evidenced low brain to plasma ratios after subcutaneous administration. Thus, these analogs are recommended for *in vitro* studies or for direct infusion into the brain.

Given the selectivity of MPX-004 and MPX-007 for GluN2A over the other GluN2 subunit containing receptors expressed in heterologous systems, these compounds may be useful for the pharmacological isolation of the GluN2A component of NMDA receptor responses in native systems. This is supported by our initial analyses of these compounds in rat pyramidal neurons in primary culture and in rat and mouse brain slice preparations. In the rat primary neuron culture studies, these compounds inhibited ~30% of the NMDA receptor mediated current, whereas a selective GluN2B NAM inhibited ~70% of the current. In the rat hippocampal slice preparation, the highest concentrations of MPX-004 tested inhibited ~60% of the total NMDA receptor-mediated fEPSP. In this preparation, a selective GluN2B NAM inhibited ~40% of the NMDA receptor-mediated fEPSP. These data suggest that these compounds may be used to selectively, and even completely, inhibit the GluN2A-mediated component of the NMDA response in native neurons. This conclusion is supported by the observation that in native tissue for which the NMDA receptor-mediated response has no GluN2A component (i.e., in visual cortex slices from *GRIN2A* KO mice), MPX-004 had no inhibitory effect. However, a point for consideration in future studies in brain slices is the potency/selectivity/solubility trade-offs between MPX-004 and MPX-007. We chose for our slice studies the more selective MPX-004; however, we reached the limit of MPX-004 solubility in the slice assay. Given its greater solubility and GluN2A potency, MPX-007 offers an alternative choice under conditions in which the possibility of low levels of GluN2B inhibition is a lesser issue.

The basis for the selectivity of TCN-201 for GluN2A subunit containing receptors has begun to be studied. Hansen et al. [26] used a domain swapping and mutagenesis approach to identify a putative binding site for TCN-201 at the interface of the GluN2A/GluN1 LBDs. TCN-201 binding at this site reduces GluN1 affinity for glycine by an allosteric mechanism to inhibit receptor activity. Glycine reciprocally reduces affinity for TCN-201, thus, this compound acts as a functionally competitive antagonist with glycine [24, 26, 31]. Based on the structural similarity of the MPX analogs to TCN-201, a similar mechanism of action seems likely. As we note, we utilized high physiological concentrations of glycine in our heterologous expression systems and found that the MPX analogs were able to potently and completely inhibit GluN2A activity. MPX-004 also inhibited the GluN2A component of the NMDA receptor response in brain slices. Thus, the MPX analogs have suitable potency and selectivity to isolate GluN2A activity even at high physiological glycine concentrations. At the level of molecular interaction with the receptor, it is interesting to note the slight difference in selectivity between MPX-004 and MPX-007 in the oocyte assay. Both of these compounds at concentrations that completely inhibit GluN2A activity have essentially no efficacy against GluN2C and D activities. However, MPX-007 evidenced a weak but concentration-dependent inhibition of GluN2B activity that was not as apparent with MPX-004. The differences between these two compounds are the halogen substituents on the phenylsulfonamide aryl ring and the presence of a methyl group on the pyrazine nucleus of MPX-007. The SAR for GluN2A potency in both of these regions was very sensitive to substitution. It will be of interest in future studies to compare the conformation of the putative TCN-201 binding site for GluN2A with that of GluN2B on the one hand and GluN2C and D on the other to gain further insight into the allosteric mechanism of action of these compounds and the regulation of the glycine binding site.

A further point for comment relates to the interactions of MPX-004 with NMDA receptors that contain a GluN2A subunit in combination with GluN2B. It is now well established that GluN2A/GluN2B heteromers comprise a significant portion of the NMDA receptor population deployed by glutamatergic principal neurons [34–36]. Hansen et al reported that TCN-201 is 4–5 fold less potent for inhibition of GluN2A/GluN2B heteromers than for GluN2A homomers expressed in oocytes [37]. In this light it is interesting that the MPX-004 concentration-response curve for inhibition of fEPSPs in the hippocampal slices was apparently monotonic and that the GluN2A- and GluN2B-mediated components to the overall NMDA receptor response were cleanly separable pharmacologically. Further studies are underway to investigate the pharmacological and molecular mechanisms of inhibition by MPX-004 and MPX-007 at GluN2A/GluN2B receptors. These studies are hoped to shed light on the molecular function of NMDA receptors and aid in the interpretation of the pharmacological effects of these compounds at the different receptor subtypes expressed in native tissues.

In summary, we identify MPX-004 and MPX-007 as new pharmacological probes of NMDA receptors containing a GluN2A subunit. The physiological importance of GluN2A-containing receptors, and their interest as therapeutic targets, cannot be overstated [6, 10]. GluN2A is the most abundant and widely expressed of the GluN2 NMDA subunits [6, 13, 14, 38]. The upregulation of GluN2A expression is thought to be an essential mechanism in the maturation of synapses during early brain development [39–43]. In adult neurons, the balance between GluN2A- and GluN2B-subunit deployment is a key determinant of the plasticity and stability of individual synapses [44–46]. Consistent with a fundamental role for GluN2A-containing receptors in development and synaptic plasticity, recent genetic analyses implicate variation in the GRIN2A gene as major risk factors for schizophrenia [47, 48] and autism [49]. Mutations in GRIN2A are strongly associated with childhood epilepsy/aphasia syndromes [50–53] and there is evidence for dysregulation of NMDA receptor subunit composition in Rett Syndrome [30, 54] and in Parkinson’s disease [55, 56]. MPX-004 and MPX-007 are highly selective pharmacological tools to further probe GluN2A physiology and involvement in these neuropsychiatric and developmental disorders.

## Materials and Methods

### Reagents

The GluN2B NAMs used in these studies were Ro 25–6981 (Tocris Bioscience, Bristol, UK; [29]) and from [57]. Unless otherwise indicated all other reagents were from Sigma-Aldrich (St. Louis, MO).

### Chemistry

Methyl 4-(aminomethyl) benzoate hydrochloride **11a** can be obtained from Sigma-Aldrich, methyl 5-(aminomethyl)pyridine-2-carboxylate **11b** from Enamine Building Blocks, methyl 5-(aminomethyl)pyridine-2-carboxylate **11c** from Aurora Building Blocks and methyl 5-chloro-6-methylpyrazine-2-carboxylate **13** from J&L PharmLab LLC. Test compounds were prepared in DMSO.

#### Synthesis of compounds 2–10

**Fig 7:**
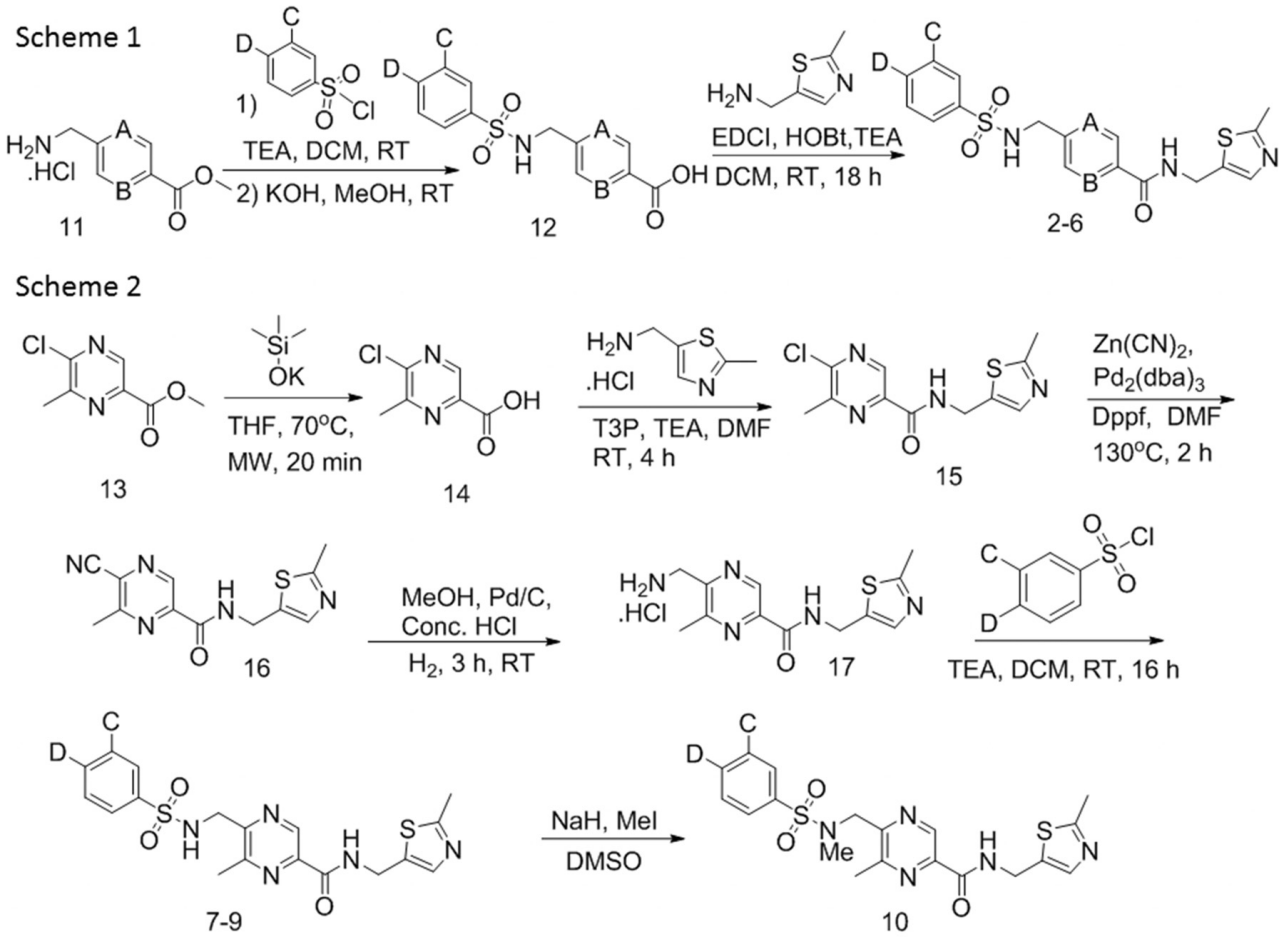
Synthetic schemes for compounds 2–10.

Benzene **2**, pyridines **3** and **6** and pyrazines **4** and **5** (Fig 7, Scheme 1) were obtained *via* a three step synthetic sequence starting with commercially available methyl 4-(aminomethyl) benzoate **11a** (A & B = C), methyl 5-(aminomethyl) picolinate **11b** (A = C; B = N) and methyl 5-(aminomethyl) pyrazine-2-carboxylate **11c** (A & B = N) respectively. Treatment of amines **11a-c** with halogen substituted benzenesulfonyl chlorides (TEA, DCM) followed by KOH mediated ester saponification cleanly afforded acids **12**. Acids **12** were converted to amides **2–6** using standard amine coupling (EDCI, HOBt, TEA, DCM) conditions.

To access methyl substituted pyrazines **7–10** (Fig 7, Scheme 2), commercially available methyl 5-chloro-6-methylpyrazine-2-carboxylate **13** was saponified (KOTMS, THF) to afford acid **14** which was coupled with (2-methylthiazol-5-yl) methanamine (T3P, DMF) to afford pyrazine **15**. Cyanide displacement (Zn(CN)2, Pd2(dba)3, Dppf, DMF) of the chloride moiety of **15** generated **16**. Nitrile reduction (H_2_, Pd/C, MeOH) of **16** afforded amine **17**. Amine **17** was cleanly converted to sulfonamides **7–9**. Methylation of sulfonamide **7** (NaH, MeI) provided **10**.

### In vitro ADME assays

#### Kinetic solubility

Compounds were prepared as stock solutions at 20 mM in DMSO and diluted in DMSO to give a range of test concentrations 100-fold greater than final. Compounds were diluted 100-fold into phosphate buffered saline and incubated during mild shaking for 90 min at room temperature. Solutions were filtered through multiscreen solubility filter plate and the filtrate analyzed for compound concentration by HPLC-UV/PDA or LC-MS/MS.

#### Stability in pooled human liver microsomes

Human liver microsomes were prepared from pooled cryopreserved human hepatocytes (HEP10™, catalog number HMCS10, lot # HuE106, Life Technologies Corporation, Grand Island, NY). According to the vendor, the liver cells from lot HuE106 were derived from tissue obtained from accredited institutions. Consent was obtained by these institutions from the donors or the donor’s legal next of kin, for use of the tissue and its derivatives for research purposes. Microsomes were diluted into 66.7 mM potassium phosphate buffer (pH 7.4) to an approximate concentration of 0.5 mg/mL of microsomal protein. Test compounds (1 μM) were pre-incubated with the microsomes for 5 min at 37°C and then the reaction initiated by addition of 10 μM NADPH. After different times up to 30 min, the reactions were quenched, clarified by centrifugation and remaining compound quantified by LC-MS/MS.

#### MDCK cell permeability

MDCK cells transfected with MDR1 were maintained in monolayer culture for 3–4 days in 24-well transporter plates. Cultures with average TEER values of 600–800 Ohm x cm^2^ were utilized for experimentation. Test compound was added to either the apical or basal membrane chamber and then efflux across the monolayer was monitored by quantifying compound with LC-MS/MS.

### Screening assays

#### Primary screening assays

HEK293 cells expressing the human GRIN1/GRIN2A genes (Chantest Catalog #: CT6120) were grown as an adherent monolayer at 37°C, 5% CO_2_ in DMEM/Ham’s F12 + 10% tetracycline-screened FBS (Hyclone SH3007.03T) and 500 μg/ml G418. At ~70–80% confluence, cells were induced by the addition of 0.3–0.4 μg/ml tetracycline in the presence of 2.5 mM ARL-15896 (AdooQ Bioscience, Irvine CA) for 15–21 hours at 37°C and then at 30°C for another 3–5 hours. To prepare the cells for an experiment, they were rinsed with Dulbecco’s PBS (Ca^2+^ and Mg^2+^ free) and detached from the flask with TrypLE™ Express (Life Technologies, Carlsbad, CA) using the manufacturer’s recommended methods. Cells collected from the flask were washed twice in Ca^2+^/Mg^2+^-free Hanks Balanced Salt Solution + 20 mM HEPES (HBSS), and counted and assessed for viability with trypan blue. Washed cells were then dye-loaded by resuspending in Fluo-8/AM calcium sensitive dye plus Component B (AAT Bioquest, Sunnyvale, CA) diluted in HBSS. To allow cells to take up fluo-8 dye, they were incubated in the dark for 15 min at 37°C, followed by 30 min at 22–25°C. After dye-loading, cells were washed and resuspended in HBSS and plated in 384-well plates (Falcon 353962; Corning, Big Flats, NY) at 20,000–30,000 cells/well in a final volume of 25 μl/well. The cell plates were centrifuged at 200 x g, for 2 minutes at 21°C, to create a monolayer of cells at the bottom of the wells.

To initiate an assay, 10 μl of test compound or buffer was added to each well of the cell plate and pre-incubated for 10 minutes in the dark. After 10 minutes, the cell plate was placed in a FDSS 6000 plate reader (Hamamatsu Photonics, Middlesex, NJ); baseline fluorescence readings were collected for 20 seconds. Next, 25 μl of agonist solution (3 μM glutamate, 3 μM glycine, and 1 mM Ca^2+^ in HBSS) was added and the change in fluorescence was recorded for 3 minutes.

Results were calculated by computing the ratio of maximum fluorescence achieved during agonist-addition phase vs. baseline fluorescence. This fluorescence ratio was then normalized between the positive control signal (glutamate + glycine) and the fully blocked negative control (10 μM MK801, Tocris Bioscience, Bristol, UK).

#### Oocyte electrophysiology

Xenopus oocytes (Xenopus 1, Dexter, MI) were initially separated from the lobes of an ovary using surgical forceps; they were then defolliculated by constant agitation at room temperature in OR-2 buffer (82.5 mM NaCl; 2.5 mM KCl; 1 mM MgCl2; 5 mM HEPES; pH 7.4) containing 2 mg/ml collagenase Type 1 (Fisher Scientific, USA) for 1.5–2 hours. The cells were then gently aspirated with a Pasteur pipette to remove the follicular tissue; this was followed by washing three times with OR-2 to remove all traces of collagenase. The oocytes were then washed in ND-96 (96 mM NaCl, 2 mM KCl, 1.8 mM CaCl2, 1 mM MgCl2, 5 mM HEPES, 50 ng/L gentamycin, 100 units/ml penicillin and 100 μg/ml streptomycin; pH 7.6) three times and this was followed by incubation in ND-96 at room temperature with mild agitation for 15 minutes; subsequently they were stored for at least 2 hours at 16°C before sorting and selection for injection. Oocytes selected for injection were incubated overnight in ND-96 at 16°C.

The human *GRIN1* gene transcript variant NR1-3 (Origene, SKU#: SC115601, Rockville, MD), *GRIN2A* gene (Origene, SKU#: SC326381), *GRIN2B* gene (B’SYS, Witterswil, Switzerland), *GRIN2C* gene (NCBI locus NM_000835, optimized for oocyte expression and synthesized by GenScript, Piscataway, NJ) and *GRIN2D* gene (NCBI locus NM_000836, sequence optimized and synthesized by GenScript) were linearized and transcribed using the mMessage mMachine kit (Life Technologies) and promoters SP6 (for GluN1) and T7 (for GluN2A-2D). cRNAs were mixed together at a ratio of 1 GluN1:2 GluN2. Each oocyte was injected with 50 nL of cRNA (~2 ng, GluN2A and GluN2B; ~38 ng, GluN2C; ~11 ng, GluN2D), and stored in ND96 (96 mM NaCl, 2 mM KCl, 1.8 mM CaCl_2_, 1 mM MgCl_2_, 5 mM HEPES, 50 ng/L gentamycin, 100 units/ml penicillin and 100 μg/ml streptomycin; pH 7.6) at 16°C for 72 h prior to recording.

Currents were recorded using a two-electrode voltage-clamp amplifier (Oocyte Clamp OC-725C; Warner Instrument Corp, Hamden, CT), and digitized using Digidata 1550A and pClamp (v.10) software (Molecular Devices, Sunnyvale, CA). Electrodes were filled with 3 M KCl and had resistances that ranged from 0.2–1 MΩ. All measurements were made at a holding potential of -40 mV. Cells were continuously perfused with oocyte recording solution containing 90 mM NaCl, 1 mM KCl, 0.5 mM BaCl_2_, and 10 mM HEPES (Ph 7.4). Glutamate and Glycine (co-agonists) were applied at respective concentrations of 7 μM and 13 μM for GluN2A, 5 μM and 3 μM for GluN2B, and 10 μM each for GluN2D. Co-agonists were applied until a plateau was reached, and test compounds were then added in the continued presence of co-agonists until a new plateau was reached. After the addition of the last test compound concentration, the oocyte was washed with oocyte recording buffer. Fraction of current remaining was calculated as [(current in presence of test compound–background current) / (co-agonist current–background current)] and IC_50_ values were computed using Prism 6.0 (Graphpad).

### Cortical neurons in primary culture

Cryopreserved E-18 rat cortical neurons (GIBCO A10840-02) were purchased from Life Technologies Italia (Monza, Italy). According to the vendor, timed-pregnant female rats were anesthetized with CO_2_ and, when unconscious, rapidly decapitated; embryos were then extracted and cortical neurons were prepared. All procedures were approved by the Institutional Animal Care and Use Committee of Biocon, Inc. (Iselin, NJ).

Cortical neurons were thawed and plated at 5x10^4^ or 10x10^4^ cells/well in 35 x 100 mm tissue culture dishes containing poly-D-lysine treated glass coverslips, and maintained in the media and conditions recommended by the manufacturer for 13–15 days. Neurons were then used in manual patch clamp experiments with intracellular solution consisting of (in mM) 120 CsF, 10 CsCl, 11 EGTA, 10 HEPES, 0.5 CaCl_2_, with pH adjusted to 7.25 with CsOH. Extracellular solution consisted of (in mM) 137 NaCl, 4 KCl, 1.8 CaCl_2_, 1 MgCl_2_, 10 HEPES, 10 D-glucose, with pH adjusted to 7.35 with NaOH. Glycine (10 μM) was added in the extracellular solution or in the NMDA solution. Cells were patch clamped in the whole-cell configuration with a holding potential of -70 mV stepping once every minute to +20 or +40 mV for 10 s. Starting 1.5 s after each voltage step, 100 μM NMDA plus 10 μM glycine was applied using rapid local perfusion (RSC-200, Bio-Logic Science Instruments, Claix, France). Currents were evaluated at peak during the NMDA application, and current inhibition was computed using Prism software (GraphPad) once steady state was achieved.

### Brain slice electrophysiology

#### Rat hippocampal slices

Multi-electrode arrays (MEA) technology was used in the recording of hippocampal slices from 3 to 4 week-old male and female Sprague- Dawley rats (Elevage Janvier, Le Genest St Isle, France). Primipara rats with their littermate were transferred from Elevage Janvier to the Neuroservice facility on post-natal day 11. The light/dark cycle maintained within the rodent facility was 12/12 hours, respectively, beginning at 7:00 AM. Temperature was maintained at 22 ± 2°C. Water and food were available *ad libitum*. Animals were euthanized by trained personnel (graduate from “experimentation animale—niveau I”) in accordance to the French and European legislations for animals care, in accordance with the Directive 2010/63/EU of the European Parliament and of the Council of 22 September 2010 in the protection of animals used for scientific purposes. Methods are essentially as described in detail in [58] and are summarized below.

The rats were sacrificed by fast decapitation, without previous anesthesia. The brain was quickly removed and soaked in ice-cold oxygenated buffer with the following composition in mM: KCl 2, NaH2PO4 1.2, MgCl2 7, CaCl2 0.5, NaHCO3 26, glucose 11, and saccharose 250. Hippocampus slices (400 μM) were cut with a McILWAIN tissue-chopper and incubated at room temperature for at least 1 h in artificial cerebrospinal fluid (ACSF) of the following composition in mM: NaCl 126, KCl 3.5, NaH_2_PO_4_ 1.2, MgCl_2_ 1.3, CaCl_2_ 2, NaHCO_3_ 25, and glucose 11, bubbled with carbogen (95% O_2_, 5% CO_2_). Slice were placed in the center of a 3D-MEA to cover the electrode field (1.4 mm^2^) and then immobilized with a netting ballast. The MEA was quickly transferred to the amplifier stage on an inverted Motic microscope, and the slice continuously perfused with oxygenated ACSF (3 ml/min at 37°C) of the following composition (in mM): NaCl 127, KCl 3.5, NaH_2_PO_4_ 1.2, MgCl_2_ 0.1, CaCl_2_ 2, NaHCO_3_ 25 and Glucose 11. NBQX (10 μM) was added to inhibit AMPA receptors. The bath was ground connected with a 1 M NaCl-Agar bridge.

All data were recorded with a MEA set-up from Multi Channel Systems MCS GmbH (Reutlingen, Germany) composed of a 4-channel stimulus generator and a 60-channel amplifier head-stage connected to a 60-channel A/D card. Softwares for stimulation, recording and analysis were the ones commercially available from Multi Channel Systems: MC Stim (2.0.3.0 release) and MC Rack (3.2.1.0 release). All of the experiments were carried out with MEA from MCS that consist of 60 electrodes spaced by 200 μM.

Following visual observation through a CCD camera, one of the electrodes identified in the CA3 field (at CA3/CA1 border) was disconnected from its amplifier and used to stimulate Schaeffer collaterals. The stimulation protocol consisted in a monopolar biphasic current pulse (−300 μA 50 μs and +300 μA 50 μs) injected every 30 sec. Simultaneous evoked field potentials were recorded from 3 to 6 electrodes in the CA1 stratum radiatum region, corresponding to the physiological propagation of the stimulus along the Schaffer collateral pathway. Field potentials were recorded from selected electrodes and sampled at 5 kHz. The NMDA component of the field potential was isolated by including 10 μM NBQX in the perfusion solution. Since recorded field potentials resulted from NMDA-mediated synaptic transmission consecutive to afferent pathway stimulation (complete inhibition of the AMPA/Kainate component due to NBQX), 30 μM D-AP5 was perfused on the slice at the end of each experiment, to validate the NMDA-mediated nature of synaptic transmission as well as to subtract background noise at individual electrode level.

MPX-004 was prepared as a 30 mM stock solution in DMSO, aliquoted and stored at -20°C. Each day, an aliquot was thawed and used to prepare 1000-fold concentrated stock solutions in DMSO (i.e. 10 mM, 3 mM, 1 mM, 0.3 mM, 0.1 mM), to reach 10 μM, 3 μM, 1 μM, 0.3 μM and 0.1 μM final concentrations in ACSF. 30 mM stock solution was 600-fold and 1000-fold diluted in ACSF to reach 50 μM and 30 μM final concentrations, respectively. DMSO final concentration was adjusted to 0.3% in all the perfused solutions. Complete solution exchange in the MEA chamber was achieved 20 s after the switch of solutions.

After collecting baseline NMDA receptor-mediated responses for 10 min, individual slices were perfused with different concentrations of MPX-004 and recordings continued for an additional 40 min, at which point inhibition was at apparent equilibrium. The NMDA fEPSP amplitude was measured as the difference between the baseline (before stimulation) and the maximal peak amplitude. Background noise at individual electrodes level was subtracted to the NMDA fEPSP amplitude. NMDA fEPSP amplitude was normalized as a function of the mean-averaged amplitude recorded over the 10-minute control period. The normalized NMDA fEPSP values of all the validated electrodes of a single slice were first averaged. Next data from the slices in the same experimental conditions were averaged. The NMDA fEPSP mean values (± SEM) were plotted versus time. Dose-inhibition curve of MPX-004 (mean inhibition of NMDA EPSP after 40 minutes) was fitted with an empirical Hill equation, yielding its IC_50_.

For MPX-004 concentrations from 0.1 to 30 μM, data was collected from 4 to 8 slices prepared from 2 or 3 different rats. For MPX-004 concentration of 50 μM, data was collected from 2 slices prepared from 1 rat.

#### Mouse visual cortex slices

Wild-type (WT) mice and *GRIN2A* knock-out (KO) male mice were bred in house at Boston Children’s Hospital. The *GRIN2A*-KO colony was derived from mice originally obtained from M. Mishina (1995, University of Tokyo, Japan). All procedures using these mice were approved by the Boston Children’s Hospital Institutional Animal Care and Use Committee. Methods used were previously described in [30] and are summarized below.

Coronal visual cortical slices (300 μM thick) were prepared from postnatal day (P)22–31 male mice following decapitation under isoflurane anesthesia. Slices were incubated at room temperature in a submerged holding chamber for at least 1 hour in artificial cerebral spinal fluid (ACSF) containing: 119 mM sodium chloride, 2.5 mM potassium chloride, 1 mM sodium dihydrogen phosphate monohydrate, 1.3 mM magnesium chloride hexahydrate, 2.5 mM calcium chloride, 26.2 mM sodium bicarbonate, 20 mM glucose, and bubbled with carbogen gas (95% oxygen/5% carbon dioxide). Slices were incubated in ACSF with MPX-004 for at least 40 minutes prior to recording. All recordings were made at room temperature in the presence of 5 μM bicuculline methiodide (Sigma, St. Louis, Missouri) to block gamma-aminobutyric acid (GABA)-ergic currents.

Layer 2/3 pyramidal neurons were selected based on morphology under infrared differential interference contrast microscopy and their identity confirmed from a brief train of action potentials recorded prior to initiating voltage-clamp recordings. Patch pipettes (6–8 MΩ) pulled from standard wall borosilicate tubing were filled with: 140 mM cesium chloride, 0.2 mM ethylene glycol tetraacetic acid (EGTA), 10 mM 4-(2-Hydroxyethyl)piperazine-1-ethanesulfonic acid (HEPES), 2 mM adenosine triphosphate-magnesium, 0.3 mM guanosine triphosphate, 5 mM QX-314, and 5 mg/mL biocytin (pH 7.2). Excitatory postsynaptic currents (EPSCs) in layer 2/3 pyramidal neurons were evoked by extracellular stimulation (0.2 msec, 16–100 μA) in layer 4 of the visual cortex using a bipolar stimulating electrode pulled from standard wall borosilicate tubing and filled with artificial cerebrospinal fluid. Voltage-clamp recordings were acquired at a 10 kHz sampling rate with an AxoPatch-1D (Axon Instruments, Union City, California) and an ITC-18 AD board (Instrutech, Mineola, New York). All data were acquired and analyzed using custom- made procedures in Igor-Pro Software (WaveMetrics, Lake Oswego, Oregon).

To estimate the relative contribution of NMDA and AMPA receptors to the synaptic current, the amplitude was measured at the time of the peak current at -100 mV for the AMPA receptor-mediated current and at the time point after the current at -100 mV had decayed to less than 5% of its peak amplitude for the NMDA receptor-mediated current. Current-voltage (I-V) plots were constructed with the current amplitudes for the NMDA and AMPA receptor-mediated components as a function of the holding potential from -100 mV to 60 mV at intervals of 20 mV. The NMDAR/ AMPAR ratio was calculated as the ratio of the slopes between 0 and 40 mV, where the slopes of the NMDA and AMPA receptor-mediated components were most linear [30]. All values are reported as mean ± SEM, unless otherwise stated.
