# Correction: MPX-004 and MPX-007: New Pharmacological Tools to Study the Physiology of NMDA Receptors Containing the GluN2A Subunit

**DOI:** 10.1371/journal.pone.0151452

**Published:** 2016-03-08

**Authors:** The PLOS ONE Staff

There is an error in affiliation 1 for authors Robert A. Volkmann, Christopher M. Fanger, David R. Anderson, Frank S. Menniti. Affiliation 1 should be:

Mnemosyne Pharmaceuticals, Inc. (now Luc Therapeutics) 400 Technology Square, Cambridge, MA 02139, United States of America
